# LoWRKY1 Alleviates PEG-Induced Membrane and Enhances Antioxidant Responses Damage in *Larix olgensis* and Tobacco

**DOI:** 10.3390/plants15203183

**Published:** 2026-10-17

**Authors:** Hao Dong, Chen Wang, Wenna Zhao, Yu Liu, Chengpeng Cui, Shujuan Li, Meng Li

**Affiliations:** State Key Laboratory of Tree Genetics and Breeding, Northeast Forestry University, Harbin 150040, China

**Keywords:** *Larix olgensis*, LoWRKY1, transcription factor, PEG-induced osmotic stress

## Abstract

Drought stress constitutes a persistent constraint on tree survival, and larch, owing to their distinctive needle morphology, deploys drought-responsive strategies that differ from those of broad-leaved trees. In this study, we identified a putative transcription factor, LoWRKY1, that is highly expressed in larch needles and induced by both polyethylene glycol (PEG)-mediated osmotic stress and abscisic acid (ABA) treatment. Heterologous overexpression of LoWRKY1 in tobacco enhanced leaf relative water content (RWC), reduced ion leakage (IL) and malondialdehyde (MDA) levels, thereby mitigating damage to the membrane system. Furthermore, LoWRKY1 was shown to promote superoxide dismutase (SOD) and catalase (CAT) activities in both tobacco and *Larix olgensis* embryonic callus cells, effectively scavenging reactive oxygen species (ROS) and improving drought tolerance. Collectively, these findings establish that LoWRKY1 plays a positive role in PEG-induced osmotic stress responses in *L. olgensis*, providing a promising candidate gene for the genetic improvement in the drought tolerance in coniferous trees. More broadly, this work highlights the value of integrating heterologous expression with conifer embryonic callus systems to dissect stress-regulatory networks in conifers, thereby offering a practical strategy to accelerate functional genomics in tree species that remain recalcitrant to stable transformation.

## 1. Introduction

Drought stress, together with associated abiotic stresses such as high temperature, accounts for substantial tree mortality annually [1]. Owing to differences in leaf morphology and wood properties, coniferous and broad-leaved trees have evolved distinct strategies for drought tolerance [2]. Broad-leaved tree species capitalize on large specific leaf area and elevated leaf nitrogen content to enhance photosynthetic efficiency, thereby enabling rapid growth and competitive advantage under resource-abundant conditions [3]. In contrast, coniferous tree species such as *Pinus koraiensis* adopt a conservative defensive strategy, characterized by reducing metabolism, enhancing protective mechanisms, and conservative carbon allocation, thereby conferring resistance to abiotic stresses such as drought [4]. Consequently, under adverse environmental conditions, coniferous species like *P. koraiensis* frequently exhibit superior drought tolerance and survival capacity relative to broad-leaved trees [5]. Moreover, coniferous tree species are characterized by extended growth cycles and slow growth rates, which represent adaptative responses to persistently nutrient-poor and drought-prone environments [6]. Drought induces oxidative stress in *Larix olgensis* and suppresses plant height growth [7]. From the perspective of long-term tree survival, elucidating the drought tolerance mechanisms of conifers is therefore of critical importance.

A high sugar-to-starch ratio enhances cold tolerance in *Juniperus virginiana* L. [8]. *Larix gmelinii* responds to abiotic stress via the trehalose metabolic pathway [9]. Under low saline–alkali stress, “Zhongshanshan 302” promoted lignin formation and enhanced cellulose crystallinity [10]. The overexpression of the ethylene transcription factor ThRAP2.1 from *T. mucronatum* × *T. distichum* in poplar promotes 1-aminocyclopropane-1-carboxylic acid (ACC) content, ACC synthase activity, and alcohol dehydrogenase (ADH) activity under flooding stress, thereby enhancing flooding tolerance [11]. Conifers primarily rely on two distinct strategies for drought tolerance: stomatal closure mediated by leaf desiccation or by abscisic acid (ABA). Both strategies depend on the coordinated evolution of leaf water supply and stomatal water loss [12]. Under water stress, *Pinus radiata* exhibits ABA-driven stomatal closure. In contrast, stomatal closure in *Callitris rhomboidea* is initiated by elevated leaf ABA levels but subsequently shifts to water-potential-driven stomatal closure, indicating interspecific variation among conifers [13]. *Sequoiadendron* seedlings exhibit greater drought tolerant than *Sequoia* with respect to water status regulation, stem embolism, root biomass partitioning, and growth sensitivity to drought [14]. Notably, *L. olgensis* has overcome technical bottlenecks in embryogenic callus proliferation and cryopreservation, establishing it as a suitable representative species for conifer research [15].

Regardless of the strategy employed to resist abiotic stress, transcription factors are indispensable. WRKYs, bHLHs, and ERFs are differentially expressed in the resin biosynthesis pathway [16]. In *Pinus yunnanensis* var. *pygmaea*, TALE, ERF, and WRKY transcription factors coordinately maintain ion homeostasis in response to phosphorus stress [17]. Exogenous auxin treatment of *Pinus massoniana* seedlings decreased the expression of the drought-positive regulator *PmalIAA27*, while increasing the expression of *PmaARF15*, which interacts with PmalIAA27 [18]. In *P. massoniana*, *PmWRKY6/10/30* are upregulated by drought, whereas *PmWRKY22* is repressed under drought conditions [19]. *P. pinaster* under drought stress exhibits high expressions of genes such as WRKY and RD22 [20]. Overexpression of LoHDZ2 in transgenic cell lines enhances peroxidase activity and reduces MDA content under drought and salt stress, thereby improving antioxidant capacity and stress tolerance [21]. In *L. olgensis*, *LoNAC5* responds to multiple phytohormones and is induced by drought and saline–alkali stress [22]. LoNAC3 negatively regulates drought and saline–alkali tolerance via the reactive ROS pathway [23].

In our previous study, we subjected *L. olgensis* to PEG-induced osmotic stress and performed transcriptome sequencing [24], revealing that the gene TRINITY_DN3766_c0_g1 exhibited a significant response to PEG-induced osmotic stress. In this study, we analyzed the sequence and domain architecture of this gene and designated it based on phylogenetic relationships. Through heterologous transformation in tobacco and overexpression in embryogenic callus of *L. olgensis*, combined with assessments of membrane system damage and ROS scavenging efficiency, we further elucidated its functional role in PEG-induced osmotic stress.

## 2. Results

### 2.1. Transcription Factor LoWRKY1 Mediates PEG-Induced Osmotic Stress Responses in L. olgensis and Responsive to ABA

**Figure 1:**
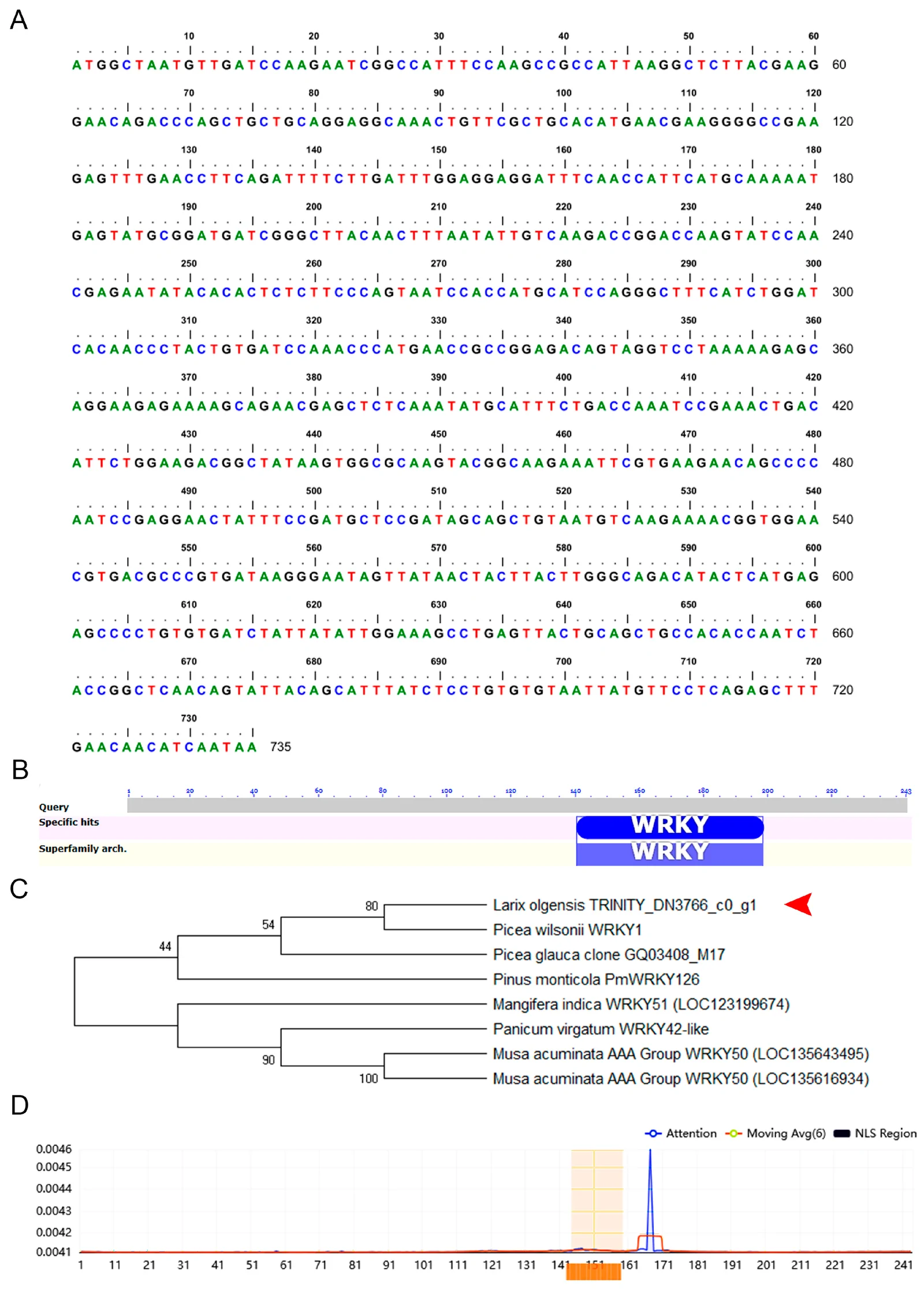
Identification of the LoWRKY1 transcription factor in *L. olgensis*. (**A**) The TRINITY_DN3766_c0_g1 gene contains a 735 bp coding sequence. (**B**) Protein domain analysis of TRINITY_DN3766_c0_g1. (**C**) Phylogenetic analysis of the TRINITY_DN3766_c0_g1 gene and its genetic relationships with other species. Red triangles indicate LoWRKY1. (**D**) Prediction of the nuclear localization signal (NLS) of the LoWRKY1 transcription factor.

In a previous study, a drought-responsive gene, TRINITY_DN3766_c0_g1, was identified in *L. olgensis* [24]. Its full-length CDS of 735 bp was subsequently cloned (Figure 1A). TRINITY_DN3766_c0_g1 encodes a predicted protein of 244 amino acids with a calculated molecular weight (Mw) of 29.77 kDa and a theoretical isoelectric point (pI) of 7.18 (Figure S1). Conserved domain analysis revealed a typical WRKY domain, indicating that this gene belongs to the WRKY transcription factor family (Figure 1B). Sequence analysis revealed that TRINITY_DN3766_c0_g1 contains a single WRKY domain with a variant WRKYGKK motif (instead of the canonical WRKYGQK) and a C2H2-type zinc-finger motif (C-X4-5-C-X22-23-H-X-H) (Figure S1). Based on the number of WRKY domains and the type of zinc-finger motif [25], TRINITY_DN3766_c0_g1 was classified into the Group II subfamily of WRKY transcription factors. BLAST (2.17.0) analysis showed that TRINITY_DN3766_c0_g1 shares high sequence similarity with WRKY proteins from *Picea* species, including *Picea glauca* clone GQ03408_M17 (86% query coverage, 83.41% identity; BT113446.1) and *Picea wilsonii* WRKY1 (85% query coverage, 81.62% identity; MW560928.1). Phylogenetic analysis using the Minimum Evolution method with the Kimura 2-parameter + Gamma distributed (K2 + G) model placed TRINITY_DN3766_c0_g1 in a conifer clade together with *Picea wilsonii* WRKY1 (bootstrap = 80) and *Picea glauca* clone GQ03408_M17 (bootstrap = 54). Based on these results, the gene was named *LoWRKY1* (Figure 1C). A putative nuclear localization signal (NLS) was further identified in the LoWRKY1 amino acid sequence, supporting its predicted role as a putative transcriptional regulator (Figure 1D). The LoWRKY1-GFP fusion was transiently expressed in poplar protoplasts, with H2A-1 as a nuclear marker. Confocal microscopy showed that LoWRKY1-GFP was exclusively localized to the nucleus and co-localized with H2A-1 (Figure S2), confirming that LoWRKY1 is a nuclear-localized protein.

**Figure 2:**
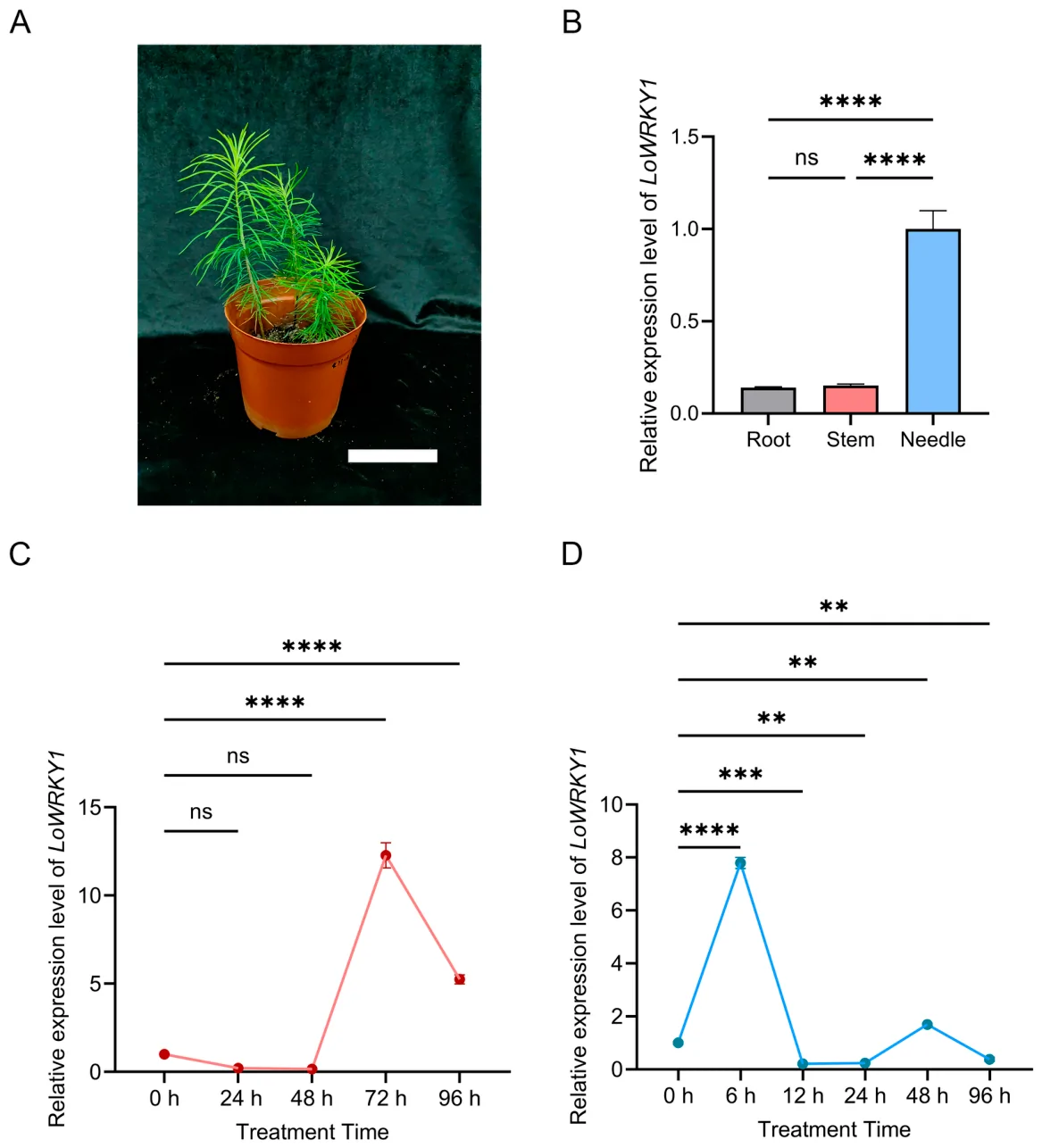
The transcription factor LoWRKY1 responds to PEG-mediated osmotic stress and ABA treatment. (**A**) Four-month-old WT *L. olgensis* plants. Scale bar: 10 cm. (**B**) Relative expression levels of *LoWRKY1* in root, stem, and needle tissues of *L. olgensis*. Data are presented as mean ± SEM (*n* = 3). Asterisks indicate significant differences among tissues (**** *p* < 0.0001; one-way ANOVA followed by Tukey’s test). “ns” indicates no significant difference between root and stem. (**C**) Changes in *LoWRKY1* expression at 0, 24, 48, 72 and 96 h under PEG_6000_ treatment. (**D**) Changes in *LoWRKY1* expression at 0, 6, 12, 24, 48 and 96 h under ABA treatment. Data are presented as mean ± SEM (*n* = 3). One-way ANOVA revealed significant differences among time points, and homogeneity of variance was confirmed by the Brown–Forsythe test. All pairwise comparisons were performed using Tukey’s test; only comparisons against 0 h are shown in the figure. Asterisks indicate significant differences compared to 0 h (** *p* < 0.01, *** *p* < 0.001, **** *p* < 0.0001); “ns” indicates no significant difference.

To characterize the drought-responsive function of *LoWRKY1*, its transcript levels were examined in roots, stems, and needles of WT *L. olgensis* plants. Expression was markedly higher in needles than in roots or stems (Figure 2A,B). Therefore, subsequent expression analyses focused on needle tissues under PEG_6000_ and ABA treatments (Figure 2C,D). Under PEG_6000_ treatment, needles were harvested at 0, 24, 48, 72, and 96 h post-treatment. *LoWRKY1* transcript levels peaked at 72 h, showing a 12.28-fold increase relative to 0 h, and remained significantly elevated (5.25-fold) at 96 h, indicating that *LoWRKY1* is strongly induced by PEG-induced osmotic stress (Figure 2C). To assess its potential responsiveness to ABA, we further quantified *LoWRKY1* expression following exogenous ABA application. Owing to the rapid responsiveness to ABA, six time points (0, 6, 12, 24, 48, and 96 h) were sampled. *LoWRKY1* transcripts were significantly upregulated as early as 6 h post-treatment, with a 7.8-fold increase over the control (Figure 2D). Collectively, these results suggest that *LoWRKY1* responds to ABA treatment in *L. olgensis*.

### 2.2. Heterologous Expression of LoWRKY1 from L. olgensis in Tobacco Enhances Drought Tolerance

**Figure 3:**
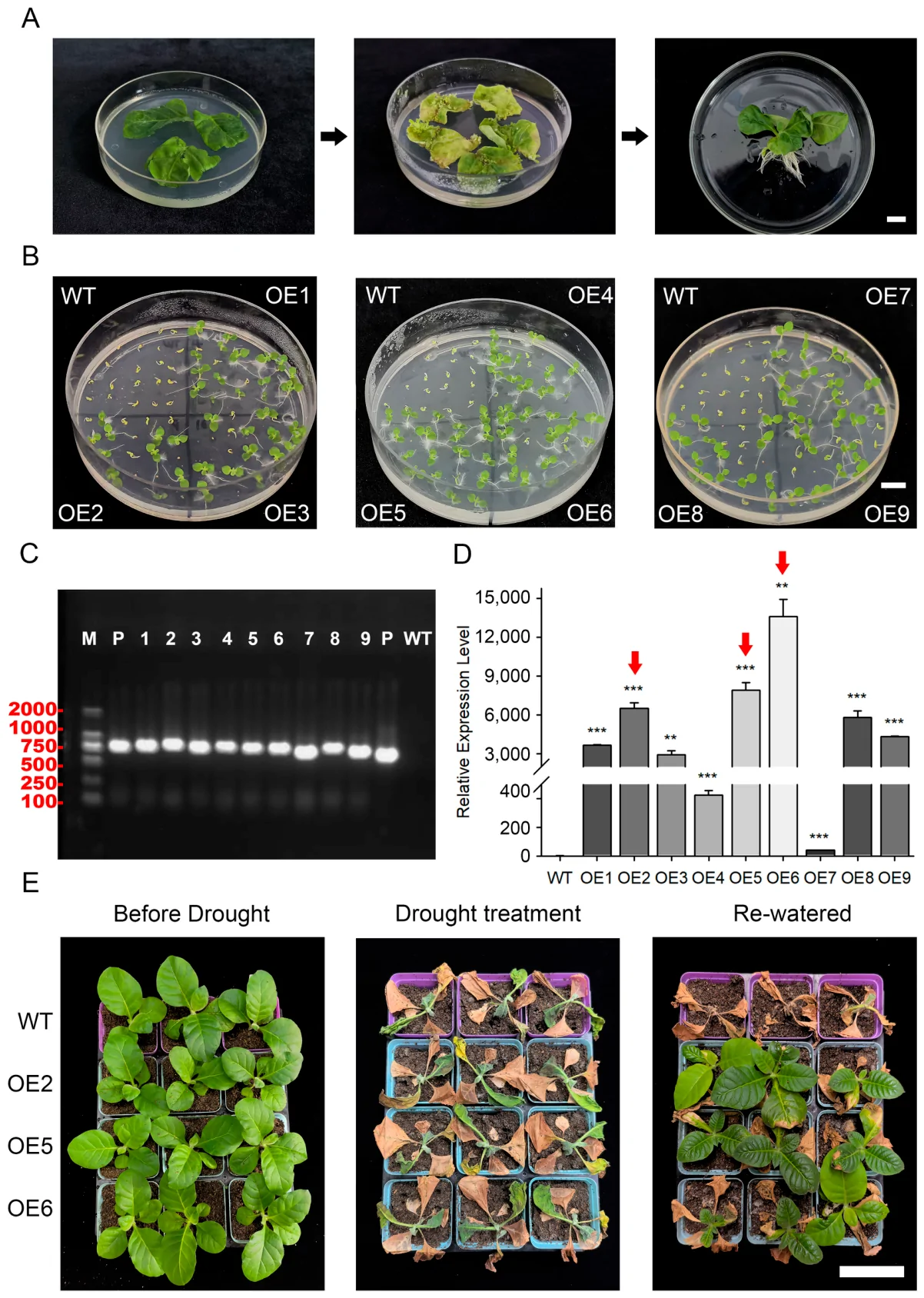
Overexpression of *LoWRKY1* enhances drought tolerance in *N. tabacum*. (**A**) *Agrobacterium*-mediated transformation of tobacco leaves was performed to obtain T1 transgenics. Scale bar: 1 cm. (**B**) Selection and culture of T2 transgenic lines. Scale bar: 1 cm. (**C**) Detection of lines with *LoWRKY1* integrated into the tobacco genome. M: DL2000 marker; P: positive control; WT: negative control. (**D**) Quantitative detection of relative expression changes in *LoWRKY1* in nine transgenic lines. Arrows indicate the three lines with the highest expression levels. Error bars represent the SEM of three individual plant. Asterisks indicate significant differences (*** *p* < 0.001; ** *p* < 0.01) as determined by Student’s *t*-test. (**E**) Drought-related phenotypes of WT and transgenic tobacco plants (OE2, OE5, OE6) before drought, after drought treatment, and after re-watering. The drought experiment was performed only once; therefore, the results are presented as a preliminary phenotypic observation. Scale bar: 10 cm.

To evaluate the functional role of *LoWRKY1* in drought stress tolerance, its coding sequence was ectopically expressed in tobacco (*Nicotiana tabacum*) via *Agrobacterium*-mediated transformation using the leaf disk method, and T1 transgenic seeds were collected (Figure 3A). Through resistance selection and PCR-based genotyping, nine independent T2 transgenic lines carrying *LoWRKY1* were obtained (Figure 3B,C). qRT-PCR confirmed that *LoWRKY1* transcripts were significantly elevated in all nine lines, which were designated *OE-LoWRKY1-#1* to *OE-LoWRKY1-#9* (Figure 3D). Among them, lines OE2, OE5, and OE6 exhibited relatively higher expression levels than other lines and were selected for further phenotypic analyses.

**Figure 4:**
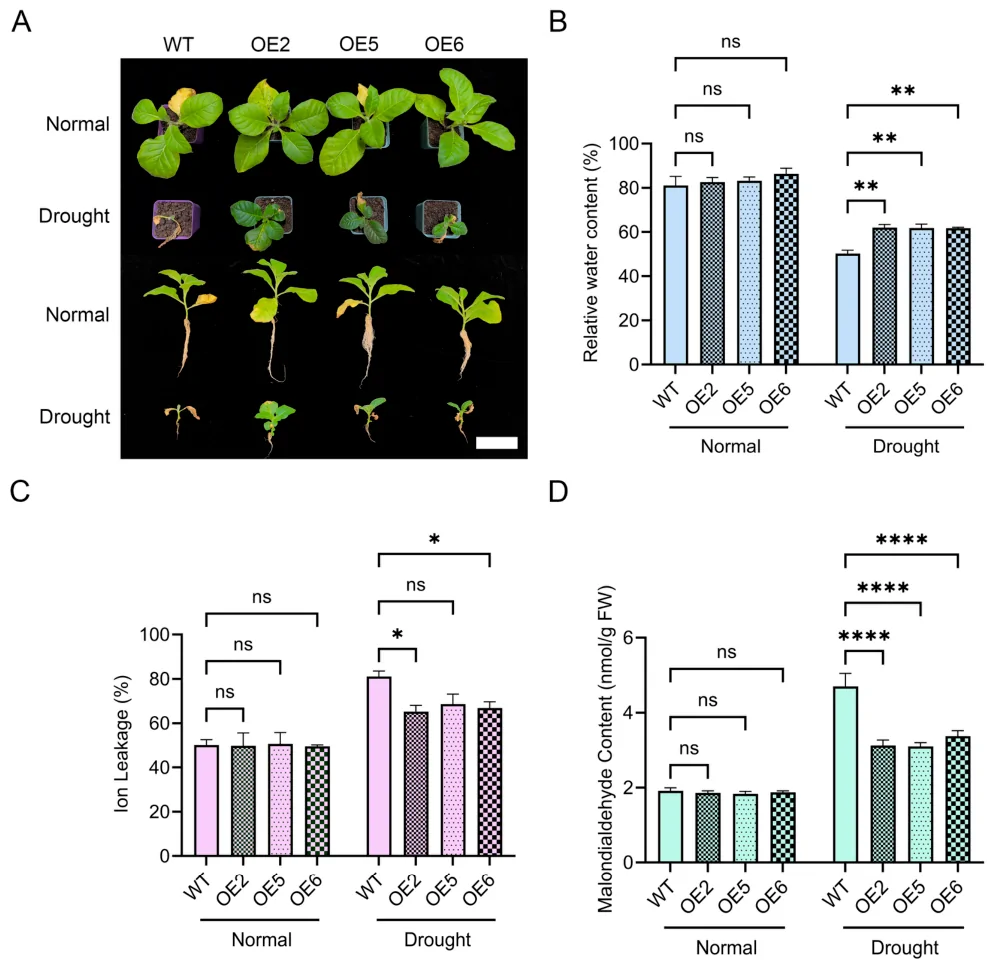
Overexpression of *LoWRKY1* alleviates drought-induced membrane damage. (**A**) Changes in OE2, OE5, OE6 and WT tobacco plants under normal and water withholding conditions. Scale bar: 10 cm. (**B**–**D**) Differences in relative water content (**B**), ion leakage (**C**) and malondialdehyde content (**D**) among OE2, OE5, OE6 and WT under normal and water withholding conditions. Data are presented as the mean ± SEM of three independent experiments (*n* = 3). Each data point represents the mean of three plants from one independent experiment. Two-way factorial ANOVA revealed a highly significant main effect of treatment (F_1, 16_ = 255.10, *p* < 0.001), confirming that Water withholding treatment significantly reduced RWC across all lines. All post hoc comparisons were corrected using Dunnett’s test adjustment. Asterisks above the drought group indicate significant differences between transgenic lines and WT under drought conditions (* *p* < 0.5; ** *p* < 0.01; **** *p* < 0.0001). And “ns” indicates no significant difference.

To assess drought tolerance, 4-week-old transgenic lines (OE2, OE5, and OE6) and WT plants were subjected to water withholding. In preliminary water withholding treatment, all three transgenic lines survived after re-watering, whereas WT plants did not, suggesting that *LoWRKY1* overexpression may improve water withholding tolerance in tobacco (Figure 3E). These results suggest that *LoWRKY1* overexpression may improve water withholding tolerance in tobacco, although this conclusion is preliminary because the experiment was performed only once. To compare drought-induced membrane damage, leaf tissues were harvested from both genotypes under normal and water withholding conditions at the time point when WT plants exhibited visible wilting (Figure 4A). Relative water content (RWC), ion leakage (IL), and malondialdehyde (MDA) content were measured (Figure 4B–D). Under normal conditions, two-way ANOVA revealed significant effects of genotype and treatment for RWC (genotype: F_3, 16_ = 5.94, *p* = 0.006; treatment: F_1, 16_ = 255.10, *p* < 0.001) and MDA (genotype: F_3, 16_ = 13.33, *p* < 0.001; treatment: F_1, 16_ = 245.04, *p* < 0.001; interaction: F_3, 16_ = 11.22, *p* < 0.001). For IL, only the treatment effect was significant (F_1, 16_ = 67.76, *p* < 0.001). Water withholding caused a significant reduction in RWC in all plants; however, RWC was significantly higher in transgenic lines (61.68–61.90% for OE2, OE5, and OE6) than in WT (50.14 ± 2.84%; Figure 4B). Although water withholding induced marked increases in IL (65.22–68.59% in OE2, OE5, and OE6 vs. 81.09 ± 4.28% in WT) and MDA (3.10–3.37 nmol/g in OE2, OE5, and OE6 vs. 4.70 ± 0.60 nmol/g in WT) in all genotypes (Dunnett’s test), the accumulation of both parameters was substantially lower in transgenic lines than in WT (Figure 4C,D). These results indicate that overexpression of *LoWRKY1* mitigates Water withholding-induced membrane damage.

**Figure 5:**
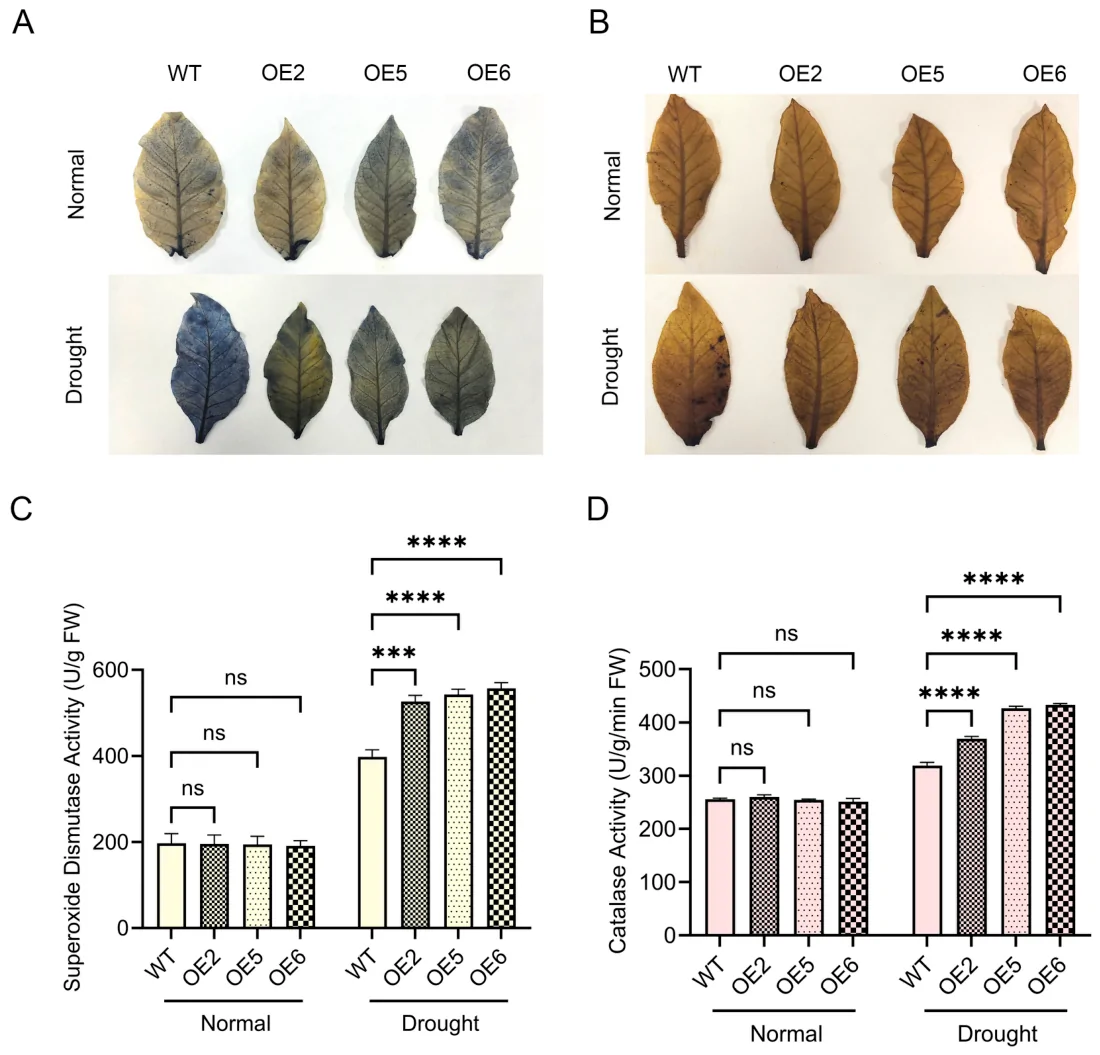
LoWRKY1 promotes ROS scavenging under water withholding. (**A**) Nitroblue tetrazolium staining was used to visualize O^2−^ accumulation in OE2, OE5, OE6 and WT plants under normal and water-withholding conditions. (**B**) 3,3′-Diaminobenzidine staining was used to compare H_2_O_2_ levels in OE2, OE5, OE6 and WT plants under normal and water-withholding conditions. (**C**,**D**) Differences in SOD (**C**) and CAT (**D**) activities in OE2, OE5, OE6 and WT plants under normal and water withholding conditions. Error bars represent the SEM among three independent biological replicates. Each data point represents the mean of three plants from one independent experiment. Asterisks indicate significant differences between transgenic lines and WT under drought conditions. (*** *p* < 0.001, **** *p* < 0.0001), as determined by two-way factorial ANOVA followed by Dunnett’s test. And “ns” indicates no significant difference.

We next examined the effect of *LoWRKY1* overexpression on ROS metabolism (Figure 5) using Nitroblue tetrazolium (NBT) staining, which detects superoxide anion (O^2−^) accumulation as blue formazan precipitates [26]. Moreover, 3,3′-diaminobenzidine (DAB) staining, which visualizes hydrogen peroxide (H_2_O_2_) as brown polymerization products [27], were performed. Under normal conditions, leaves of both transgenic lines and WT exhibited minimal staining with both NBT and DAB (Figure 5A). Under water withholding, WT leaves showed intense blue NBT staining, whereas the three transgenic lines displayed visibly lighter staining. DAB staining revealed brown spots in both WT and transgenic leaves under water withholding, and the transgenic lines showed visibly lighter DAB staining than WT, which is consistent with reduced H_2_O_2_ accumulation (Figure 5B). These results suggest that *LoWRKY1* overexpression is associated with reduced ROS accumulation under water withholding.

To determine whether *LoWRKY1* is associated with enhanced ROS scavenging, we measured the activities of superoxide dismutase (SOD) and catalase (CAT) [28,29] in leaves of transgenic and WT plants. SOD and CAT activities were measured in WT and transgenic lines (OE2, OE5, OE6) under control and drought conditions (Figure 5C,D). Two-way ANOVA revealed significant effects of genotype, treatment, and their interaction for both enzymes (*p* < 0.01). Under normal conditions, CAT and SOD activities were comparable among all genotypes. However, under water withholding, both SOD and CAT activities increased significantly in all lines and were significantly higher in transgenic lines than in WT (Dunnett’s test). For CAT, OE5 and OE6 showed the highest activity, followed by OE2, while WT showed the lowest. For SOD, OE6 showed the highest activity, followed by OE5 and OE2, while WT showed the lowest. These results are consistent with a model in which elevated SOD activity converts O^2−^ to H_2_O_2_, which is subsequently detoxified by increased CAT activity (Figure 5C,D).

Collectively, these findings demonstrate that ectopic expression of *LoWRKY1* improves water withholding-related physiological responses in tobacco, including reduced membrane damage and enhancing antioxidant enzyme activities.

### 2.3. The Response Pattern of LoWRKY1 in L. olgensis Callus Was Consistent with That Observed in Tobacco

**Figure 6:**
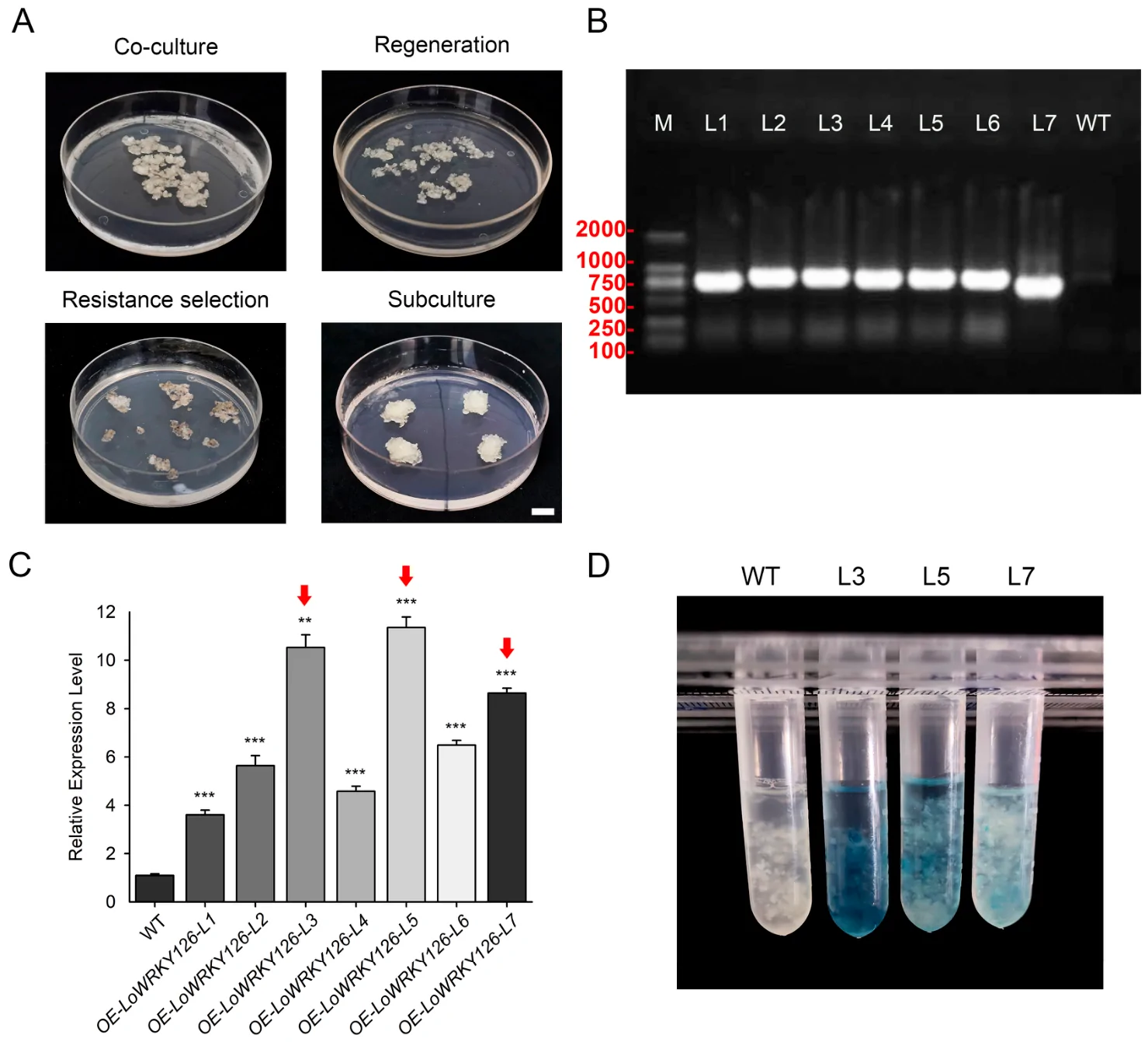
Generation of *LoWRKY1*-overexpressing transgenic lines in embryogenic callus of *L. olgensis*. (**A**) Representative images of *Agrobacterium*-mediated transformation of *L. olgensis* embryogenic callus, including co-culture, regeneration, resistance selection, and subculture stages. Scale bar: 1 cm. (**B**) PCR amplification of the *LoWRKY1* gene in putative transgenic callus lines (L1–L7) and WT. WT as negative control; M: DL2000 marker. (**C**) Relative expression levels of *LoWRKY1* in transgenic callus lines and WT, determined by qRT-PCR. The red arrows indicate the three lines (L3, L5, and L7) with the highest expression levels, which were selected for subsequent experiments. Data are presented as mean ± SEM (*n* = 3). Asterisks indicate significant differences compared to WT (*** *p* < 0.001; ** *p* < 0.01) as determined by Student’s *t*-test. (**D**) GUS staining of WT and selected transgenic lines (L3, L5, L7).

Because heterologous expression systems may not fully recapitulate the native regulatory networks of genes in their original species [30], we generated transgenic *L. olgensis* embryogenic callus via *Agrobacterium*-mediated transformation (Figure 6A). Seven resistant callus lines, designated as *OE-LoWRKY1 L1* to *L7*, were identified by PCR (Figure 6B), and *LoWRKY1* transcript levels were determined by qRT-PCR. Lines *OE-LoWRKY1-L3*, *OE-LoWRKY1-L5*, and *OE-LoWRKY1-L7* exhibited significantly higher expression levels than the others (Figure 6C). GUS staining also revealed blue coloration in the L3, L5, and L7 lines, indicating successful expression of the LoWRKY1-GUS fusion protein (Figure 6D). Thus, lines *OE-LoWRKY1-L3*, *OE-LoWRKY1-L5*, and *OE-LoWRKY1-L7* were selected for further analysis.

**Figure 7:**
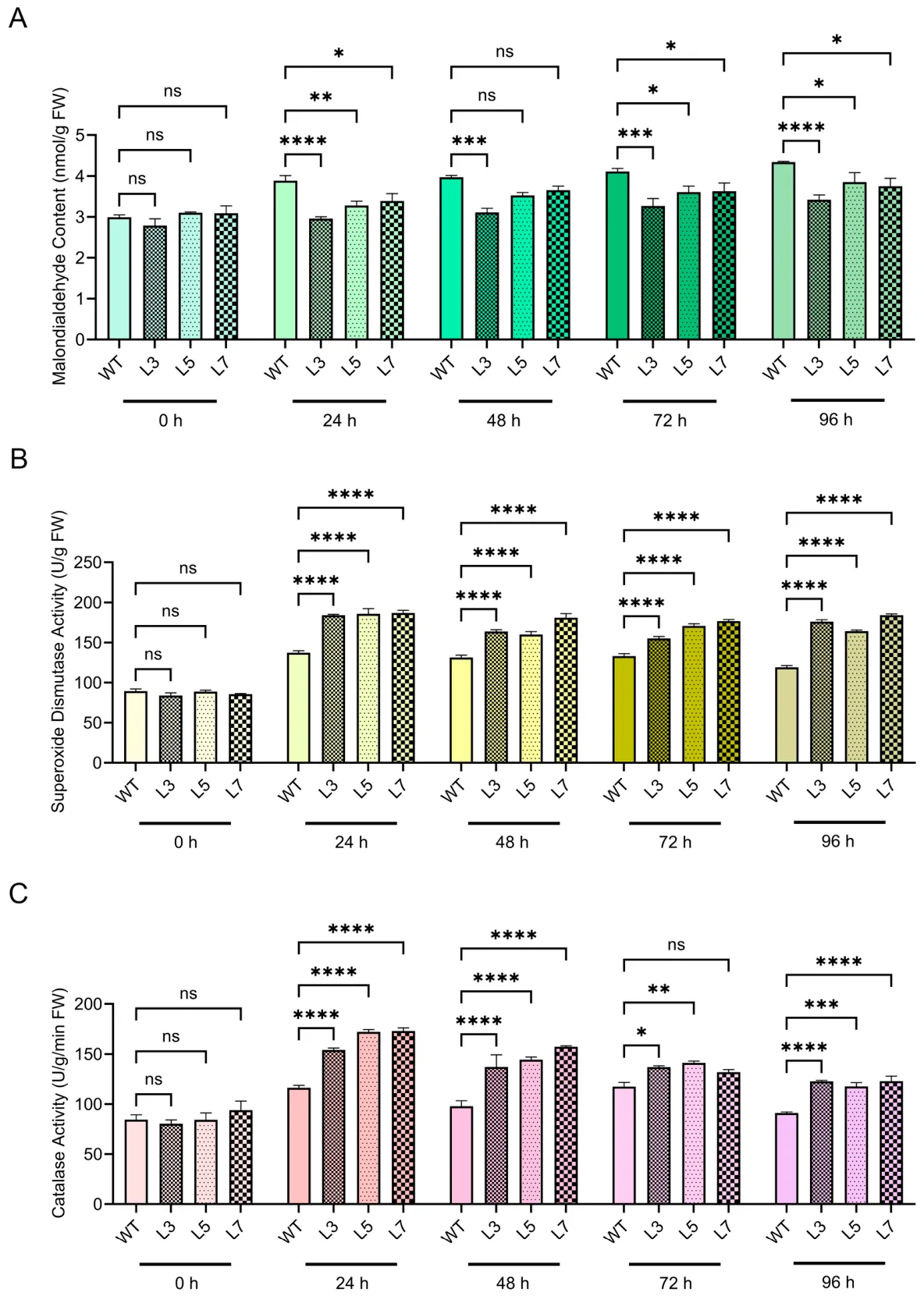
LoWRKY1 reduces membrane damage and enhances ROS scavenging capacity in embryogenic callus of *L. olgensis*. (**A**–**C**) Differences in MDA content (**A**), SOD activity (**B**) and CAT activity (**C**) between *OE-LoWRKY1-L3*, *OE-LoWRKY1-L5*, *OE-LoWRKY1-L7* and WT callus. Error bars represent the SEM of three independent biological replicates. Asterisks indicate significant differences between transgenic lines and WT at the corresponding time points (* *p* < 0.05, ** *p* < 0.01, *** *p* < 0.001, **** *p* < 0.0001), as determined by linear mixed-effects models followed by Dunnett’s test. And “ns” indicates no significant difference.

Consistent with the observations in transgenic tobacco, the transgenic callus lines (*OE-LoWRKY1-L3*, *OE-LoWRKY1-L5*, and *OE-LoWRKY1-L7*) exhibited significantly lower MDA accumulation than WT under PEG-induced osmotic stress (Figure 7A), indicating that LoWRKY1 effectively alleviates membrane lipid peroxidation in its native species context. For CAT and SOD, no significant differences were observed among genotypes at 0 h. From 24 h onward, L3, L5, and L7 all showed significantly higher CAT and SOD activities than WT (Dunnett’s test), with L7 maintaining the highest levels at 96 h (Figure 7B,C), consistent with the antioxidant enzyme response observed in the heterologous tobacco system. CAT, SOD, and MDA levels were measured in WT and transgenic lines (L3, L5, L7) at 0, 24, 48, 72, and 96 h (Figure 7A,C). Data were analyzed using linear mixed-effects models with time and genotype as fixed factors, including their interaction, and with experimental replicate as a random effect. These results indicate that *LoWRKY1* overexpression is associated with enhanced antioxidant enzyme activities and reduced membrane damage in *L. olgensis* under PEG-induced osmotic stress.

## 3. Discussion

Conifer and broad-leaved trees are evolutionarily distant, and stress tolerance mechanisms identified in broad-leaved species cannot be directly extrapolated to conifers [31,32]. Therefore, species-specific studies in coniferous trees are essential. Here, building on our previous work, we identified the drought-responsive gene TRINITY_DN3766_c0_g1 in *L. olgensis*, based on its domain characteristics and phylogenetic analysis, designated it as *LoWRKY1* (Figure 1B,C and Figure S1). Overexpression of *LoWRKY1* in tobacco reduced membrane damage under water withholding (Figure 4), and enhanced ROS scavenging capacity (Figure 5). The similar response pattern observed in embryogenic callus of *L. olgensis* (Figure 7A–C) further supports the involvement of LoWRKY1 in osmotic stress-related stress responses.

WRKY family members are involved in sugar metabolism during heteroblastic change in conifer needles [33], and are predicted to play important roles in lateral bud development [34]. In addition, transcription factors such as WRKY and NAC also function in the formation of parenchyma cells from vessel cells [35]. A total of 83 WRKY family members were identified in *P. monticola* [36], whereas only 31 WRKY genes have been identified in *Pinus massoniana* Lamb [37]. Although both species belong to the genus *Pinus*, the loss of transcription factors may contribute to functional divergence. LoWRKY1 is a putative transcription factor (Figure S1) and responds to PEG-induced osmotic stress (Figure 2C), highlighting the role of WRKY family genes in stress tolerance in conifers. The high expression of *LoWRKY1* in needles suggests its specific function in leaves (Figure 2B). Moreover, LoWRKY1 in needles responds to ABA treatment which is associated with stomatal opening and closure [38]. However, the function of LoWRKY1 in stomatal closure was not explored in this study. *LoWRKY1* is highly expressed in larch needles but is also expressed in roots and stems (Figure 2B), indicating organ-specific differences in transcriptional regulation. The coordinated and tissue-specific regulatory mechanisms among different larch tissues also represent a promising direction for future research.

RWC is a sensitive indicator of water deficit perception in larch. When RWC falls below 60%, tissue damage occurs in *Pinus ponderosa* var. *scopulorum Dougl. ex Laws* [39]. The higher RWC in *LoWRKY1*-overexpressing tobacco plants under water withholding compared with WT plants likely protects them from damage (Figure 4B), as further evidenced by the lower IL and MDA contents (Figure 4C,D). In our experiments, the SOD and CAT activities measured in *L. olgensis* were much lower than those measured in tobacco (Figure 5C,D and Figure 7B,C), suggesting that SOD and CAT activities are lower in larch than in tobacco. This is consistent with previous studies on *Pinus pinaster* and *Quercus robur*, in which the activities of SOD, CAT, and APX in conifers were found to be 2- to 4-fold lower than those in *Quercus robur* [40], indicating that conifers generally show lower activities of antioxidant enzymes than broad-leaf species. In addition, our data shows an association between *LoWRKY1* overexpression and increased SOD/CAT activities (Figure 5C,D and Figure 7B,C), but do not demonstrate that LoWRKY1 directly regulates antioxidant genes. Future work will focus on identifying downstream target genes, testing whether LoWRKY1 directly binds their promoters, and validating direct DNA binding, which will help clarify the precise molecular mechanism by which LoWRKY1 functions in osmotic stress.

Additionally, the NBT and DAB staining data presented in this study are qualitative and are shown only as representative images (Figure 5A,B); in addition, these analyses were not conducted in *L. olgensis* callus. Although the staining patterns are consistent with reduced ROS accumulation in transgenic lines, they do not provide a quantitative measure of superoxide or H_2_O_2_ levels. Future experiments combining quantitative ROS measurements with histochemical staining will be needed to confirm these observations.

The primary reason that research on conifers lags behind that on broad-leaved trees is the lack of a well-established genetic transformation system, with gene function validation generally relying on heterologous transformation in exogenous plant species [41]. The native-species validation in this study was performed only in *L. olgensis* embryogenic callus, in which MDA, SOD, and CAT were measured (Figure 7), resulting in the conclusions regarding the function of LoWRKY1 in the native species which need to be considered preliminary. The male strobilus-specific promoters of *Pinus radiata D. Don* (*PrCHS1*, *PrLTP2*, *PrMC2*, and *PrMALE1*) were fused with the GUS reporter gene, and their spatiotemporal expression patterns were analyzed in *Arabidopsis*, revealing the issue of functional conservation of *cis*-elements in heterologous hosts [42]. We note that LoWRKY1 was expressed as a C-terminal GUS fusion (Figure 3B and Figure 6A), and the possibility that the GUS tag may affect its folding, localization, or activity cannot be fully excluded. Furthermore, an empty-vector control was not included in this study, so the effects of transformation, antibiotic selection, and tissue culture cannot be fully separated from those of LoWRKY1. Further validation using untagged LoWRKY1 and an empty-vector control will be pursued in future work.

Four NAC transcription factors were identified from *Pinus densiflora*. Transient overexpression in tobacco and *Arabidopsis* leaves induced tracheid-like secondary cell wall deposition, demonstrating that these conifer transcription factors possess conserved regulatory functions in angiosperms [43]. Overexpression of the *Cunninghamia lanceolata* (Lamb.) Hook ClCOBL1 gene in tobacco resulted in altered leaf adaxial–abaxial polarity and short, swollen corolla tubes, demonstrating that this conifer gene is involved in the regulation of cell wall deposition and cell expansion direction [44]. We were unable to obtain complete transgenic larch plants. However, in this study, we attempted to obtain transgenic plants using larch embryogenic callus cells (Figure 3A–D and Figure 6A–D). By relying on callus proliferation and cryopreservation methods to continuously maintain the transgenic materials [15], we achieved gene function validation in the native plant species to the greatest extent possible (Figure 7).

In this study, LoWRKY1 was identified as a WRKY transcription factor based on the presence of a WRKY, a C2H2-type zinc-finger motif (Figure S1). We further confirmed its nuclear localization using an LoWRKY1-GFP fusion transiently expressed in poplar protoplasts (Figure S2), supporting its classification as a nuclear-localized transcription factor. However, the present study does not identify the direct downstream targets of LoWRKY1, and no DNA-binding assays (e.g., EMSA, ChIP-qPCR, or Y1H) were performed. Therefore, the mechanistic statements in this manuscript are limited to its involvement in the osmotic stress response and its ABA-responsive expression, rather than a demonstrated direct regulatory role.

## 4. Conclusions

In this study, we characterized the role of LoWRKY1 in PEG-induced osmotic stress. Overexpression of *LoWRKY1* in tobacco improved osmotic stress-related physiological responses, including higher relative water content and antioxidant enzyme activities (CAT and SOD), and lower IL and MDA content under water withholding treament. In embryogenic callus of *L. olgensis*, *LoWRKY1* overexpression enhanced PEG-induced osmotic stress responses, with higher CAT and SOD activities and lower MDA accumulation compared with WT. *LoWRKY1* expression was also responsive to ABA treatment. However, the present study does not establish a complete ABA-dependent molecular mechanism. The direct downstream targets of LoWRKY1 and the precise contribution of ABA signaling to its function remain to be determined in future work.

## 5. Materials and Methods

### 5.1. Plant Materials

Seeds of *L. olgensis* collected from the Qingshan Seed Orchard in Linkou County, Heilongjiang Province, were rinsed with running water for 3–5 days. Plump and settled seeds were selected and sown in nutrient soil (peat soil: vermiculite = 1:1, *v*/*v*). Seedlings were cultured under conditions of 25 ± 1 °C, a photoperiod of 16 h/8 h (light/dark), a light intensity of 80 μmol·m^−2^·s^−1^, and a relative air humidity of 75%. During cultivation, the seedlings were covered with plastic film to maintain moisture, and the film was removed every 3 days for ventilation and watering to maintain appropriate soil moisture and prevent waterlogging. Thirty days after sowing, when the needles were fully expanded, uniformly growing seedlings were selected and transplanted into larger pots and cultured for an additional period.

*N. tabacum* seeds were surface-sterilized by sequential washing with sterile water twice, 75% ethanol for 40 s, sterile water three times, 20% sodium hypochlorite for 10 min, and sterile water three times. The sterilized seeds were then sown on 1/2 MS medium and cultured in a growth room under a photoperiod of 16 h/8 h (light/dark), 25 ± 1 °C, and 75% relative humidity. After 10 days, healthy seedlings were selected and transplanted into sterilized nutrient soil (peat soil: vermiculite = 1:1, *v*/*v*). The soil-grown seedlings were maintained under the same environmental conditions until they reached the 4- to 6- leaf stage, at which point they were used for water-withholding treatments.

### 5.2. Vector Construction

Needles of *L. olgensis* were collected, frozen in liquid nitrogen, and ground to a fine powder. Total RNA was extracted using the Plant RNA Extraction Kit (OMEGA, Norcross, GA, USA) according to the manufacturer’s instructions. Genomic DNA was removed, and RNA was reverse-transcribed into cDNA using the reverse transcription kit (TaKaRa, Kusatsu, Shiga, Japan). The *LoWRKY1* gene was amplified using this cDNA as a template with the gene-specific primers as follows: *LoWRKY1*-F: CGGGATCCATGGCTAATGTTGATCCAAGAATCG; *LoWRKY1*-R: ACGCGTCGACTTGATGTTGTTCAAAGCTCTGAGG. The LoWRKY1-GUS construct was generated by fusing the full-length CDS of LoWRKY1 upstream of the GUS reporter gene into the VB191103-GUS plant expression vector, creating a translational fusion (*LoWRKY1*-GUS). The construct is driven by the CaMV 35S promoter. The resulting *LoWRKY1-GUS* plasmid was then transformed into *Agrobacterium tumefaciens* strain GV3101 competent cells using the liquid nitrogen freeze–thaw method [45]. The transformed *Agrobacterium* was used for genetic transformation of tobacco leaves and embryogenic callus of *L. olgensis*.

### 5.3. Prediction of LoWRKY1 Protein Domains

The conserved domains of LoWRKY1 were predicted using the online tool at https://www.ncbi.nlm.nih.gov/Structure/cdd/wrpsb.cgi (accessed on 10 May 2025). The probability of NLS in the LoWRKY1 sequence was predicted using the tool at https://www.novopro.cn/tools/nls-signal-prediction.html (accessed on 12 May 2025). The theoretical isoelectric point (pI) and molecular weight (Mw) of LoWRKY1 were predicted using the Compute pI/Mw tool on the ExPASy server (https://web.expasy.org/compute_pi/ (accessed on 20 June 2024)).

### 5.4. Phylogenetic Analysis

The BLAST tool (https://blast.ncbi.nlm.nih.gov/Blast.cgi (accessed on 15 August 2026)) was used to identify species and sequences with similarity to *LoWRKY1*. A total of 9 sequences were used for phylogenetic analysis. Multiple sequence alignment was performed using MUSCLE 3.8.31, followed by manual adjustment. Phylogenetic analysis was performed using MEGA X (version 10.0.5) based on the Minimum Evolution method with the Kimura 2-parameter + Gamma distributed (K2 + G) model, which was selected as the best-fit model according to the Bayesian Information Criterion (BIC = 1624.254). Gaps and missing data were treated with partial deletion (95% site coverage cutoff), and all codon positions were included. Bootstrap analysis was performed with 1000 replicates, and bootstrap values are shown at the corresponding nodes.

### 5.5. PEG and ABA Treatment

Four-month-old *L. olgensis* seedlings were treated with 50 mg/L ABA and 30% (*w*/*v*) PEG_6000_. ABA was applied by both spraying and drenching, with samples collected at 0, 6, 12, 24, 48, and 96 h after treatment. PEG_6000_ was applied by drenching, with samples collected at 0, 24, 48, 72, and 96 h after treatment. PEG_6000_ was dissolved in water to a final concentration of 30% (*w*/*v*), corresponding to an osmotic potential of approximately −0.53 MPa at 25 °C [46]. The PEG solution was applied by pouring 400 mL into each pot to ensure full contact with the larch roots. As a mock treatment, the same volume of medium without PEG was used. ABA was applied by both spraying and drenching to ensure that the hormone reached both the shoot and root systems. ABA was dissolved in a small volume of ethanol and diluted with distilled water to a final concentration of 50 mg/L, with the final ethanol concentration not exceeding 0.1% (*v*/*v*). The concentration was selected based on preliminary dose-testing experiments. The ABA solution was applied by both spraying and drenching: 50 mL was sprayed onto the leaves, and 400 mL was poured into each pot. Control plants received the same volume of solution without ABA. Three biological replicates were performed for each treatment. Needles were collected, rinsed with deionized water, blotted dry with filter paper, wrapped in aluminum foil, and immediately frozen in liquid nitrogen.

Total RNA was extracted using the Plant RNA Extraction Kit (OMEGA, Norcross, GA, USA) according to the manufacturer’s instructions. RNA quality was assessed using a NanoDrop 2000 spectrophotometer (Thermo Scientific, Wilmington, DE, USA), with A260/A280 ratios between 1.8 and 2.1 and A260/A230 ratios above 2.0. First-strand cDNA was synthesized from 1 μg of total RNA using the PrimeScript RT reagent Kit (TaKaRa, Kusatsu, Shiga, Japan). Quantitative real-time PCR was performed using the TransStart Top Green qPCR SuperMix kit (TransGen Biotech, Beijing, China) according to the manufacturer’s instructions on a Bio-Rad CFX96 system (Bio-Rad, Hercules, CA, USA). Each sample was run in two technical replicates. Amplification specificity was confirmed by melting curve analysis. Relative expression was calculated using the 2^−ΔΔCt^ method, with *tubulin* gene was used as the internal reference gene. The primers used are listed as follows: RT-*LoWRKY1*-F: GTGAAGAACAGCCCCAATCC; RT-*LoWRKY1*-R: GATCACACAGGGGCTCTCAT; RT-*tubulin*-F: AGGCCGTTTCAATGCTCGG; RT-*tubulin*-R: GAAGCAGACATATTCGCATG.

### 5.6. Agrobacterium-Mediated Transformation of Tobacco

The transformation of tobacco leaves with *LoWRKY1* via the leaf disk method was performed as described in a previous report [47]. Briefly, *Agrobacterium* harboring LoWRKY1-GUS was cultured to an OD_600_ of 0.5–0.6. Cells were collected by centrifugation and resuspended in infiltration medium. Fresh leaves from approximately 30-day-old tobacco plants were cut into small pieces and infected with the *Agrobacterium* suspension. After co-cultivation in the dark for 2–3 days, the leaf pieces were transferred to medium containing 500 mg/L cefotaxime to eliminate *Agrobacterium*, and then cultured on selection medium supplemented with 500 mg/L cefotaxime and 20 mg/L hygromycin. The medium was refreshed every 10 days until resistant shoots emerged.

The resistant shoots were transferred to rooting medium containing 500 mg/L cefotaxime and 20 mg/L hygromycin and cultured until root formation. After the roots were well-established, the plants were transplanted into nutrient soil and grown to maturity; these plants were designated as the T0 transgenic lines. Seeds harvested from T0 plants (T1 generation) were surface-sterilized and sown on tobacco seed germination medium containing 25 mg/L hygromycin, and cultured in the growth room. After 10 days, healthy seedlings were selected and transplanted into autoclaved nutrient soil. Upon maturity, seeds were harvested (T2 generation). The T2 seeds were subjected to the same sterilization, sowing, and transplantation procedure to obtain T2 generation transgenic tobacco plants.

### 5.7. Water Withholding Treatment of Tobacco Plants

T2 transgenic lines OE2, OE5, OE6 and WT tobacco plants were watered to saturation and then subjected to drought treatment in the greenhouse. Plants were grown in square pots (7 × 7 × 8 cm), each filled with approximately 200 g of a peat soil: vermiculite = 1:1, *v*/*v*, with one plant per pot. Each line (WT, OE2, OE5, OE6) was represented by three independent pots. Before drought treatment, all pots were watered to saturation. Plants were maintained in a greenhouse at 25 ± 2 °C under a 16 h light/8 h dark photoperiod, with 60 ± 5% relative humidity and a light intensity of 80 μmol m^−2^ s^−1^. Pots were arranged in a randomized complete block design and repositioned every two days. At 18 days after water withholding treatment, the growth status of the plants was recorded by photography. Stress progression was assessed by visual scoring of leaf wilting and by measuring MDA, CAT, RWC, and other parameters at the end of the treatment.

For the survival analysis, the remaining plants were then rewatered, and their survival status was observed 3 days later. The drought survival experiment was performed only once, and therefore the survival data are presented as a preliminary phenotypic observation rather than a statistically supported conclusion.

### 5.8. Measurement of Physiological Parameters

The complete experiment was independently repeated three times. In each independent experiment, three plants per line were used, and the average of the three plants was calculated as one data point for that independent experiment. Therefore, each data point represents the mean of three plants from one independent experiment, and the statistical analysis was performed on the three independent experiments (*n* = 3). Technical measurements from the same plant were averaged before analysis and were not treated as biological replicates. Data are presented as the mean ± SEM of three independent experiments.

Leaf samples from the same position were collected from both normally watered and drought-treated plants, and their fresh weight was recorded as Wf. The leaves were then immersed in deionized water for 24 h. After removing surface water with filter paper, the turgid weight was recorded as Wt. The leaves were subsequently dried in a forced-air oven at 80 °C to constant weight, and the dry weight was recorded as Wd. The relative water content (RWC) of the leaf tissues was calculated using the formula: RWC = ((Wf − Wd)/(Wt − Wd)) × 100%.

Leaf samples (0.1 g) were cut into small pieces, placed in centrifuge tubes, and soaked in 10 mL of deionized water for 8 h. The electrical conductivity of the solution was measured and recorded as T1. The samples were then boiled in a water bath for 30 min, cooled to room temperature, and the conductivity was measured again as T2. Ion leakage was calculated using the formula: IL = (T1/T2) × 100%, and normalized to tissue fresh weight (0.1 g). Biological replicates were independently measured (*n* = 3).

Tissues including tobacco leaves or callus (0.1–0.2 g) were ground to a fine powder in liquid nitrogen and transferred to a 2 mL centrifuge tube. Then, 1.5 mL of 10% TCA was added, and the mixture was incubated at 4 °C for 30 min, followed by centrifugation at 11,000 rpm for 10 min. An aliquot of 1 mL of the supernatant was mixed with 1 mL of 0.6% TBA, heated in a boiling water bath for 15 min, and immediately cooled on ice. After centrifugation at 12,000 rpm for 20 min, the absorbance of the supernatant was measured. MDA content was measured using the thiobarbituric acid (TBA) method as previously described [48], with modifications. Absorbance was recorded at 450, 532, and 600 nm, and MDA content was calculated using the formula, MDA content = (6.45 × (A532 − A600) − 0.56 × A450) × V × 1000/W, where V is the extraction volume (mL) and W is the sample fresh weight (g). A standard curve was generated using 1,1,3,3-tetramethoxypropane to calibrate the assay. MDA content is expressed as nmol/g FW.

Leaf samples from the same position of each line were collected and immersed in freshly prepared NBT and DAB staining solutions, respectively, and incubated overnight at 28 °C in the dark. The staining solution was then removed, and 80% (*v*/*v*) ethanol was added. The samples were placed in a forced-air oven at 80 °C for decolorization, during which the ethanol was replaced as needed. After decolorization, the staining patterns on the leaves were observed and photographed. The NBT and DAB staining data are qualitative and are presented as representative images.

The frozen leaf or callus samples were ground to a fine powder in liquid nitrogen, then homogenized in 0.1 mol/L PBS on ice. The homogenate was centrifuged at 4000 rpm for 10 min, and the supernatant was collected as the test solution. SOD and CAT activities were determined using the corresponding assay kits (Nanjing Jiancheng Bioengineering Institute, Nanjing, China) according to the manufacturer’s instructions. SOD activity was measured by the WST-1 method and expressed as U/g FW. CAT activity was measured by monitoring the decomposition of H_2_O_2_ at 240 nm and expressed as U/g/min FW.

### 5.9. Genetic Transformation of Embryogenic Callus of L. olgensis

*Agrobacterium* harboring LoWRKY1-GUS was cultured to an OD_600_ of 0.7. The bacterial culture was transferred to sterile 50 mL centrifuge tubes, and the cells were collected by centrifugation at 4 °C and 8000 rpm for 10 min. The supernatant was discarded, and the pellet was washed with an appropriate volume of resuspension buffer and adjusted to an OD_600_ of 0.5. Fresh embryogenic callus was immersed in the infection solution and gently agitated for 20 min. The callus was then separated from the infection solution, and excess bacterial suspension was removed by blotting with sterile filter paper. The treated callus was transferred to co-cultivation medium and incubated at 25 °C in the dark for 2–3 days until visible bacterial colonies appeared on the tissue surface. The callus was then washed with sterile water containing 500 mg/L cefotaxime for decontamination, blotted dry with sterile filter paper, and cultured on recovery medium at 25 °C in the dark for 7–14 days. The embryogenic callus was then transferred to selection medium and subcultured every 10 days for three rounds of selection. Finally, the obtained *OE-LoWRKY1* transgenic callus was placed on proliferation medium and maintained through regular subculture.

### 5.10. GUS Staining

Three transgenic lines with relatively high expression levels (*OE-LoWRKY1-L3*, *OE-LoWRKY1-L5*, *OE-LoWRKY1-L7*) and WT were submerged in the GUS staining solution, subjected to vacuum infiltration for 40 min, and incubated at 37 °C in the dark for 12 h. GUS staining of the embryogenic callus was then observed and documented.

### 5.11. PEG-Induced Osmotic Stress Treatment of Embryogenic Callus of L. olgensis

Freshly proliferated WT and *OE-LoWRKY1-L3*, *OE-LoWRKY1-L5*, *OE-LoWRKY1-L7* callus (0.2 g each) were spread evenly on PEG_6000_-containing medium. For each cell line, three biological replicates were performed. Samples were collected at 0 h (non-stressed control), 24 h, 48 h, 72 h, and 96 h after treatment, labeled, and stored at −80 °C for the subsequent measurement of MDA content, SOD activity, and CAT activity as described previously.

### 5.12. Subcellular Localization of LoWRKY1

The coding sequence of LoWRKY1 was fused to the N-terminus of GFP under the control of the CaMV 35S promoter. The resulting construct (35S: LoWRKY1-GFP) and the control (35S: GFP) were transiently expressed in poplar protoplasts via PEG-mediated transformation. After incubation at 25 °C for 12 h in the dark, GFP fluorescence was observed using confocal laser scanning microscopy LSM800 (Zeiss, Oberkochen, Germany). H2A-1 was used as a nuclear maker.

### 5.13. Statistical Analysis

All statistical analyses were performed using SPSS 19.0.0 (IBM, Armonk, NY, USA). For gene expression analysis, differences between transgenic lines and WT were compared using a two-tailed Student’s *t*-test. For the tobacco experiment, data were analyzed using a two-way factorial ANOVA with genotype and treatment as fixed factors, including their interaction. Each data point represents the mean of three plants from one independent experiment, and the analysis was based on three independent experiments (*n* = 3). Post hoc pairwise comparisons were performed using Dunnett’s test. For the callus time course, data for SOD, MDA, and CAT were analyzed using linear mixed-effects models with time and genotype as fixed factors, including their interaction, and with experimental replicate as a random effect. Post hoc pairwise comparisons were performed using Dunnett’s test. Error bars represent the SEM. The value of *p* < 0.05 was considered statistically significant.

## Data Availability

The *LoWRKY1* sequence generated in this study has been deposited in GenBank under accession number QB136207. All other data supporting the findings of this study, including primer sequences, gene expression data, physiological measurements, and image data, are available within the article and its Supplementary Materials. Transgenic plant and callus materials generated in this study are available from the corresponding author upon reasonable request.

## Supplementary Materials

The following supporting information can be downloaded at https://www.mdpi.com/article/10.3390/plants15203183/s1, Figure S1: Sequence analysis of the WRKY and C2H2 domain of LoWRKY1 proteins; Figure S2: Subcellular localization of LoWRKY1 in poplar protoplasts.

## References

[1] McDowell N.G., Sapes G., Pivovaroff A., Adams H.D., Allen C.D., Anderegg W.R.L., Arend M., Breshears D.D., Brodribb T., Choat B. (2022). Mechanisms of woody-plant mortality under rising drought, CO_2_ and vapour pressure deficit. Nat. Rev. Earth Environ..

[2] Singh S.P., Gumber S., Singh R.D., Li T., Pandey R. (2025). A global comparative analysis of drought responses of pines and oaks. Forests.

[3] Ho M.C.N., Kalyuzhny M., Umaña M.N., Ostling A.M. (2025). Leaf allocation improves predictability of interspecific growth rates in a broadleaf deciduous temperate forest. Ecology.

[4] Tian J., Zhang H., Wang A., Wu J., Rosner S., Zhu K., Cai R., Yuan F. (2025). Unravelling the combined effects of drought and nitrogen addition on carbon assimilation and reserves in Korean pine saplings. Front. Plant Sci..

[5] Li B., Li W., Saha S., Ma X., Liu Y., Wu H., Zhang P., Shen H.-L. (2025). Hierarchic branch morphology, needle chlorophyll content, and needle and branch non-structural carbohydrate concentrations (NSCs) imply young *Pinus koraiensis* trees exhibit diverse responses under different light conditions. Horticulturae.

[6] Ramírez-Valiente J.A., González-Martínez S.C., Robledo-Arnuncio J.J., Matesanz S., Anadon-Rosell A., Martínez-Vilalta J., López R., Cano-Martín F.J. (2025). Genetically based trait coordination and phenotypic plasticity of growth, gas exchange, allometry, and hydraulics across the distribution range of *Pinus pinaster*. New Phytol..

[7] Zhang L., Yan S., Zhang S., Yan P., Wang J., Zhang H. (2021). Glutathione, carbohydrate and other metabolites of *Larix olgensis* A. Henry reponse to polyethylene glycol-simulated drought stress. PLoS ONE.

[8] Harbol S.C., Long R.W., Medeiros J.S. (2024). Juniperus virginiana sourced from colder climates maintain higher ratios of soluble sugars to starch during cold acclimation. Tree Physiol..

[9] Zhang X., Zhang Y., Yan M., Si Q., Yang F., Liu B., Shan J., Wang J., Wang R., Shao Y. (2025). Trehalose metabolism contributed to abiotic stress tolerance of *Larix gmelinii* seedlings. Plant Physiol. Biochem..

[10] Zhao S., Xing S., Peng J., Yu C., Pan B., Shi J. (2026). The effects of saline-alkali stress on *Taxodium* “Zhongshanshan302”: Insights into wood formation and metabolic adaptation. Physiol. Plant.

[11] Xuan L., Qi H., Wang Z., Zhou Z., Yang Y., Yu C., Hua J. (2025). Related to APETALA2.1, an interacting protein of alcohol dehydrogenase 1, is crucial for the flooding-stress responses in *Taxodium* hybrids. Int. J. Biol. Macromol..

[12] Brodribb T.J., McAdam S.A., Jordan G.J., Martins S.C. (2014). Conifer species adapt to low-rainfall climates by following one of two divergent pathways. Proc. Natl. Acad. Sci. USA.

[13] Brodribb T.J., McAdam S.A. (2013). Abscisic acid mediates a divergence in the drought response of two conifers. Plant Physiol..

[14] Ambrose A.R., Baxter W.L., Wong C.S., Næsborg R.R., Williams C.B., Dawson T.E. (2015). Contrasting drought-response strategies in California redwoods. Tree Physiol..

[15] Wang C., Zhao W., Liu Y., Dong H., Ning Y., Cui C., Zhang H., Li M., Li S. (2025). A cryopreservation and regeneration protocol for embryogenic callus of *Larix olgensis*. Plants.

[16] Bai Q., He B., Cai Y., Lian H., Zhang Q. (2020). Transcriptomic and metabolomic analyses reveal several critical metabolic pathways and candidate genes involved in resin biosynthesis in *Pinus massoniana*. Mol. Genet. Genom..

[17] Xiao F., Zhao Y., Wang X., Jian X., Yang Y. (2026). Integrative physiological and transcriptomic insights into the low-phosphorus stress response of *Pinus yunnanensis* var. *pygmaea*. BMC Plant Biol..

[18] Li L., Li Y., Quan W., Ding G. (2023). Effects of PmaIAA27 and PmaARF15 genes on drought stress tolerance in *Pinus massoniana*. BMC Plant Biol..

[19] Sun S., Chen H., Yang Z., Lu J., Wu D., Luo Q., Jia J., Tan J. (2022). Identification of WRKY transcription factor family genes in *Pinus massoniana* Lamb. and their expression patterns and functions in response to drought stress. BMC Plant Biol..

[20] Pérez-Oliver M.A., González-Mas M.D.C., Renau-Morata B., Arrillaga I., Sales E. (2023). Heat-priming during somatic embryogenesis increased resilience to drought stress in the generated maritime pine (*Pinus pinaster*) plants. Int. J. Mol. Sci..

[21] An P., Qin R., Zhao Q., Li X., Wang C., Cao Q., Zhang H., Zhang L. (2022). Genetic transformation of LoHDZ2 and analysis of its function to enhance stress resistance in *Larix olgensis*. Sci. Rep..

[22] Cao Q., Du J., Yin M., Wang C., Zhang T., Zhao Q., Liu L., Zhang H., Zhang L. (2025). Isolation and expression pattern analysis of *Larix olgensis* LoNAC5: LoNAC5 acts as a positive regulator of drought and salt tolerance. Plants.

[23] Cao Q., Hao J., Zhang T., Liu L., Xu D., Wang C., Zhao Q., Zhang H., Zhang L. (2024). Isolation and functional analysis of the *Larix olgensis* LoNAC3 transcription factor gene. BMC Plant Biol..

[24] Zhao W., Dong H., Cui C., Wang C., Liu Y., Ning Y., Zhang H., Li M., Li S. (2025). Integrated transcriptomic and WGCNA identify drought-responsive TFs in *Larix olgensis*. BMC Plant Biol..

[25] Chen C., Chen X., Han J., Lu W., Ren Z. (2020). Genome-wide analysis of the WRKY gene family in the cucumber genome and transcriptome-wide identification of WRKY transcription factors that respond to biotic and abiotic stresses. BMC Plant Biol..

[26] Bournonville C.F.G., Díaz-Ricci J.C. (2011). Quantitative determination of superoxide in plant leaves using a modified NBT staining method. Phytochem. Anal. PCA.

[27] Clarke J. (2009). Phenotypic analysis of *Arabidopsis* mutants: Diaminobenzidine stain for hydrogen peroxide. Cold Spring Harb. Protoc..

[28] McCord J.M., Fridovich I. (1969). Superoxide dismutase. An enzymic function for erythrocuprein (hemocuprein). J. Biol. Chem..

[29] May D.W. (1901). Catalase, a new enzym of general occurrence. Science.

[30] Thanthrige N., Lawrence N., Craik D.J. (2026). Biotechnological approaches for producing therapeutic peptides in plants. npj Sci. Plants.

[31] De La Torre A.R., Piot A., Liu B., Wilhite B., Weiss M., Porth I. (2020). Functional and morphological evolution in gymnosperms: A portrait of implicated gene families. Evol. Appl..

[32] Hu X.G., Zhou S.S., Yang Y., Liu H., Anil S., Wang Q., Zhao W., Gao Q., El-Kassaby Y., Wang T. (2020). Transcriptome-wide identification and profiling of miRNAs in a stress-tolerant conifer *Sabina chinensis*. J. Biosci..

[33] Zhao Y., Tu J., Wang H., Xu Y., Wu F. (2023). Transcriptomic and targeted metabolomic unravelling the molecular mechanism of sugar metabolism regulating heteroblastic changes in *Pinus massoniana* seedlings. Plant Physiol. Biochem..

[34] Chen H., Tan J., Liang X., Tang S., Jia J., Yang Z. (2021). Molecular mechanism of lateral bud differentiation of *Pinus massoniana* based on high-throughput sequencing. Sci. Rep..

[35] Guo Y., Jiao L., Wang J., Ma L., Lu Y., Zhang Y., Guo J., Yin Y. (2024). Analyses of high spatial resolution datasets identify genes associated with multi-layered secondary cell wall thickening in *Pinus bungeana*. Ann. Bot..

[36] Liu J.J., Ekramoddoullah A.K. (2009). Identification and characterization of the WRKY transcription factor family in *Pinus monticola*. Genome.

[37] Yao S., Wu F., Hao Q., Ji K. (2020). Transcriptome-wide identification of WRKY transcription factors and their expression profiles under different types of biological and abiotic stress in *Pinus massoniana* Lamb. Genes.

[38] Hsu P.K., Dubeaux G., Takahashi Y., Schroeder J.I. (2021). Signaling mechanisms in abscisic acid-mediated stomatal closure. Plant J..

[39] Vance N.C., Zaerr J.B. (1991). Influence of drought stress and low irradiance on plant water relations and structural constituents in needles of *Pinus ponderosa* seedlings. Tree Physiol..

[40] Schwanz P., Polle A. (2001). Differential stress responses of antioxidative systems to drought in pendunculate oak (*Quercus robur*) and maritime pine (*Pinus pinaster*) grown under high CO_2_ concentrations. J. Exp. Bot..

[41] Zhao H., Zhang J., Zhao J., Niu S. (2024). Genetic transformation in conifers: Current status and future prospects. For. Res..

[42] Höfig K.P., Moyle R.L., Putterill J., Walter C. (2003). Expression analysis of four *Pinus radiata* male cone promoters in the heterologous host *Arabidopsis*. Planta.

[43] Kim M.H., Tran T.N.A., Cho J.S., Park E.J., Lee H., Kim D.G., Hwang S., Ko J.H. (2021). Wood transcriptome analysis of *Pinus densiflora* identifies genes critical for secondary cell wall formation and NAC transcription factors involved in tracheid formation. Tree Physiol..

[44] Gao Y., Bian L., Shi J., Xu J., Xi M., Wang G. (2013). Expression of a conifer COBRA-like gene ClCOBL1 from Chinese fir (*Cunninghamia lanceolata*) alters the leaf architecture in tobacco. Plant Physiol. Biochem..

[45] Höfgen R., Willmitzer L. (1988). Storage of competent cells for *Agrobacterium* transformation. Nucleic Acids Res..

[46] Michel B.E., Kaufmann M.R. (1973). The osmotic potential of polyethylene glycol 6000. Plant Physiol..

[47] Horsch R.B., Fry J.E., Hoffman N.L., Eichholtz D., Rogers S.G., Fraley R.T. (1985). A simple and general method for transferring genes into plants. Science.

[48] Heath R.L., Packer L. (1968). Photoperoxidation in isolated chloroplasts. I. Kinetics and stoichiometry of fatty acid peroxidation. Arch. Biochem. Biophys..

