# Supplementary material for: LoWRKY1 Alleviates PEG-Induced Membrane and Enhances Antioxidant Responses Damage in Larix olgensis and Tobacco
Source: Plants (Basel). 2026 Oct 17;15(20):3183. doi: 10.3390/plants15203183 (PMC13709238; doi:10.3390/plants15203183)
Supplement: Supplementary file 1 [file plants-15-03183-s001.zip › plants-4598316-supplementary.pdf]

Supplementary Figures

LoWRKY1 alleviates PEG-induced membrane and enhances antioxidant responses damage in *Larix olgensis* and tobacco

Hao Dong †, Chen Wang †, Wenna Zhao, Yu Liu, Chengpeng Cui, Shujuan Li * and Meng Li *

State Key Laboratory of Tree Genetics and Breeding, Northeast Forestry University, Harbin 150040, China

* Correspondence: limeng7708@163.com (M. L.); lishujuan@nefu.edu.cn (S. L.).

† These authors contributed equally to this work

Compute pI/Mw - Results

Theoretical pI/Mw (average)

Sequence:

```
        10         20         30         40         50         60
MANVDPRIGH FQAAIKALTK EQTQLLQEAN CSLHMNEGAE EFEPSDFLDL EEDFNHSCKN

        70         80         90        100        110        120
EYADDRAYNF NIVKTGPSIQ REYTHSLPSN PPCIQGFHLD HNPTVIQTHE PPETVGPKKS

       130        140        150        160        170        180
RKRKAERALK YAFLTKSETD ILEDGYKWRK YGKKFVKNSP NPRNYFRCSD SSCNVKKTVE

       190        200        210        220        230        240
RDARDKGIVI TTYLGRHTHE SPCVIYYIGK PELLQLPHQS TGSTVLQHLS PVCNYVPQSF

EQHQ
```

Theoretical pI/Mw: 7.18 / 27994.41

Figure S1. Sequence analysis of the WRKY and C2H2 domain of LoWRKY1 proteins. The conserved WRKYGKK motif and C2H2-type zinc-finger motif are marked with red lines.

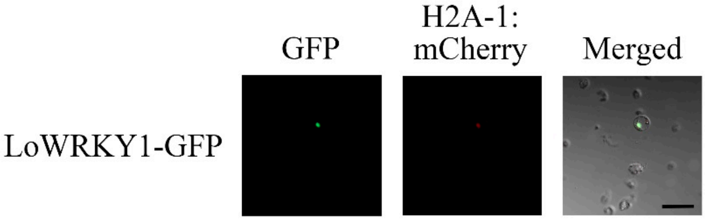

Figure S2. Subcellular localization of LoWRKY1 in poplar protoplasts. LoWRKY1-GFP and free GFP were transiently expressed in poplar protoplasts via PEG-mediated transformation. GFP fluorescence (green), H2A-1: mCherry (red), and merged images are shown. LoWRKY1-GFP was exclusively localized to the nucleus. Scale bar = 200 μm.
